# Sensitivity of Ostracods to U, Cd and Cu: The Case of *Cypridopsis vidua*

**DOI:** 10.3390/toxics10070349

**Published:** 2022-06-24

**Authors:** Liang Chen, Zheng Huo, Chi Su, Yong Liu, Wei Huang, Shan Liu, Peng Feng, Zhixin Guo, Zhihua Su, Haiyang He, Qinglin Sui

**Affiliations:** 1 School of Resource Environment and Safety Engineering, University of South China, Hengyang 421001, China; 2010000502@usc.edu.cn (L.C.); huozzz96@gmail.com (Z.H.); s954776587@gmail.com (C.S.); 1996000514@usc.edu.cn (W.H.); 2012000526@usc.edu.cn (S.L.); fengpeng@usc.edu.cn (P.F.); guozhixin@usc.edu.cn (Z.G.); 2 Hunan Key Laboratory of Rare Metal Minerals Exploitation and Geological Disposal of Wastes, Hengyang 421001, China; 3 Hunan Province Engineering Technology Research Centre of Uranium Tailings Treatment Technology, Hengyang 421001, China; 1992001751@usc.edu.cn; 4 School of Management Science, Guizhou University of Finance and Economics, Guiyang 550025, China; 201201063@mail.gufe.edu.cn

**Keywords:** ostracod, susceptibility, combined toxicity, MIXTOX model

## Abstract

The development of uranium mines has been necessary to obtain abundant and scarce uranium resources, but they also bring inevitable radioactive contamination to the surrounding soil, rivers and lakes. This paper explores the sensitivity of *Cypridopsis vidua* to the radioactive element uranium and the heavy elements cadmium and copper with single and combined acute toxicity experiments and combined toxicity model predictions. The results from the single toxicity experiments showed that the degree of toxic effects was cadmium > copper > uranium. The combined toxicity experiments showed that the compound toxicity of U-Cd and U-Cu was higher than the weakest component and lower than the strongest component, whereas the compound toxicity of Cd-Cu was higher than either of its components. When the overall proportion of a more toxic metal was increased, its mixed toxicity also increased, and vice versa. Combined toxicity predictions showed that the U-Cd combination was best described by the concentration additive (CA) model, the independent action (IA) model was more applicable to the Cd-Cu combination, and the most applicable model for the U-Cu combination changed depending on the concentration gradient. The acute toxicity data from this study provide a reference for the development of wastewater discharge standards for uranium mines, enriches the data related to the toxicity of uranium for ostracods and deepens the understanding of the threat of uranium pollution to aquatic ecosystems.

## 1. Introduction

Uranium resources for the national nuclear industry and defence are mainly obtained by mining. The mining process is accompanied by the production of radionuclides and heavy metal elements, which will cause unavoidable pollution of the soil, rivers and lakes around the mines and create a serious threat to the local ecology, agricultural systems and human health [1,2]. Neiva et al. [3] found contamination of surrounding surface water with harmful elements such as U, As, Cd, Cr and Cu at the abandoned Mortórios uranium mine in Portugal, where the levels of U, Cd and Cu reached 100, 13.4 and 24 µg·L^−1^, respectively. Trenfield et al. [4] detected U concentrations as high as 2.08 mg·L^−1^ in surface water contaminated with uranium near the World Heritage Area in northern Australia. Yi et al. [5] investigated contaminant element levels in surface water near uranium mining areas in Jiangxi Province, China and found that both U and Th levels far exceeded background values for local surface water. As a result, and as a matter of urgency, it is important to carry out environmental remediation of contaminated areas. Environmental contamination evaluation is an important part of remediation work and is performed throughout this process. Environmental contamination evaluation is mainly divided into physico-chemical evaluation [6,7,8], which is a routine evaluation of the degree of pollution hazards based on the content of relevant chemical components in samples, and biological evaluation [9,10], which is often used as an important supplement to physical and chemical evaluation, and can provide direct information on the effects of pollutants on the health of organisms and the ecosystems in which they are located. As the concept of environmental protection has become more important, the requirements for environmental assessment have also improved, and the combination of physico-chemical and biological assessments provides a more comprehensive and accurate reflection of the health of an environment [11].

Ostracods are an important part of aquatic ecosystems belonging to the phylum Arthropoda Crustacea [12]. They are recognized as good environmental indicator organisms with a series of characteristics, such as small size, large number, rich diversity, wide distribution, low research cost, easy collection and laboratory culture, and are widely used in toxicity experiments to monitor and evaluate heavy metal pollution [13,14,15,16,17,18]. Sivalingam et al. [19] exposed ostracods to sediment samples from five freshwater lakes in India to assess their ecotoxicity and showed that mortality ranged 6–23% for lightly polluted countryside lakes and 28–88% for urban lakes with high metal pollution due to anthropogenic activities. Anandakumar et al. [20] investigated the effects of industrial effluents on the predation rate of the ostracod *Cypridopsis* sp. with an analysis of sample data from February 2019 and found that species diversity was significantly affected by polluted wastewater rather than common seasonal variation. Species richness and species evenness were highly correlated. Tan et al. [21] found that the biophase of ostracods was highly correlated with physical environmental variables, and the diversity of ostracods was low in lagoon silt sediments where heavy metal concentrations reached toxic levels.

Currently, the majority of domestic and international water quality standards are formulated by only considering the contribution of individual pollutants without paying attention to interactions between pollutants and its related effects [22,23,24]. For most organisms, the toxicity criteria for individual pollutants may underestimate the toxic effects of combined exposure. Moreover, studies on ostracods have mainly focused on taxonomy [25,26,27], and few toxicological studies and evaluations of ecological health status have used ostracods. Therefore, we conducted single and combined acute toxicity experiments and made combined toxicity model predictions for three contaminants, U, Cd and Cu, which are commonly found in uranium mining process by ostracods [28,29]. Studying the effects of these metals on aquatic organisms could revise emission standards for the uranium mining industry to better protect the surrounding ecological environment, as well as improve the understanding of the damage to aquatic ecosystems caused by radioactive and heavy metal pollution.

## 2. Materials and Methods

### 2.1. Material

The sources of U, Cd and Cu used in the experiments were UO_2_(NO_3_)_2_·6H_2_O (analytical purity, >99%), CdCl_2_·2.5H_2_O (analytical purity, >99%) and CuSO4·5H_2_O (analytical purity, >99%), respectively. Storage solutions were prepared to a concentration of 1 mg·L^−1^ using ultrapure water before the experiment, and subsequently diluted by adding aeration water to achieve the desired concentrations. For accuracy testing, five concentrations of these solutions (6.25/12.5/25/50/100 µg·L^−1^) were selected for chemical analysis of all three elements, and the analytical values were 95–105% of the nominal concentrations.

The experimental organism was *Cypridopsis vidua* (*C. vidua*), which is one of the few species of freshwater ostracods with strong swimming ability. It is often found in pools with dense aquatic vegetation, dislikes anoxic environments, and lives in groups or swims under floating leaves to escape the effects of ultraviolet light. Juveniles start to appear in March each year, with a low number in winter, and can reproduce for several generations per year in the laboratory. For this study, organisms were collected from lakes and rivers around Hengyang area, Hunan Province, China, identified and classified according to their hard and soft body characteristics, and cultured in the laboratory for a long time (temperature 25 °C, light-dark ratio 16 h: 8 h, pH 7.5 ± 0.2, dissolved oxygen > 5 mg·L^−1^). *C. vidua* were pre-cultured for 1 week before the experiment, fed *Chlorella pyrenoidosa* and aeration water was added regularly. Feeding was stopped 24 h before the start of the experiment, and adult individuals of similar size and vigour were selected for the experiment.

### 2.2. Method

#### 2.2.1. Single Acute Toxicity Test

The 4-d direct exposure acute toxicity test method using the hydrostatic exposure method was performed according to standard operating procedures [30]. The minimum non-lethal concentration and the maximum total lethal concentration were obtained in a pilot study, and five concentration gradients were set up with equal ratios of each element. Three parallel groups were set up for each concentration along with a blank control group. Water temperature, pH, hardness and dissolved oxygen content were measured before and after the experiment. Ten ostracods were placed in each treatment group with 50 mL of the experimental solution and were not fed during the exposure period. Mortality curves were obtained for each time point by observing, recording and removing dead individuals from each treatment group at 2, 4, 8, 12, 24, 48, 72 and 96 h. Death was defined as the absence of life activity in the worms within 15 s of shaking the beaker. Single experimental data were analysed by probabilistic regression using IBM SPSS Statistics 26.0 (Norman H. Nie et al., Chicago, IL, USA) software to obtain each lethal dose at 96 h (96 h-LC_x_) and applied to the design of toxicant dose levels for the combined toxicity experiments, with results based on nominal concentrations.

#### 2.2.2. Combined Acute Toxicity Test

The combined toxicity experiments were also conducted using a 4-d direct exposure acute toxicity test method and hydrostatic exposure. The experimental concentration design was based on the equivalent effect concentration ratio method (EECR) and the direct equipartition ray method (EquRay). These two methods are suitable for toxicity assessments in a binary mixture system and can effectively characterize the mixing ratio and concentration level diversity in the system with multiple representative rays that reflect the toxicity pattern more comprehensively. The concentration design for the combined toxicity experiments was based on the lethal concentration values from the single toxicity experiments. Firstly, the 96 h-LC_10_, LC_30_ and LC_50_ values for U, Cd and Cu were selected according to the equivalent effect fixed concentration ratio method, and three equivalent concentration ratios, EE10, EE30 and EE50, were constructed by pairing two groups. Secondly, the 96 h-LC_50_ values for two of the three metals were selected and connected according the direct homogeneous ray method, and this experiment was set up with five mixture concentration points according to the design, producing a total of five mixed effect concentration ratios from Eq1 to Eq5. Each group of binary mixtures contained a total of eight concentration ratio designs. Finally, on the basis of keeping the concentration ratio design fixed for each group, the experiment was carried out by selecting the appropriate dilution factor from high to low for 12 concentration gradients according to the equal-effect concentration ratio method. The results of the combined experiments were fitted to concentration–response curves using six nonlinear functions in MIXTOX, and the combined toxicity was predicted with the assistance of CA and IA reference models, where the two were compared and analysed to determine the interaction mode for the combined toxicity effect of the mixture.

(1) Fixed ratio ray design (FRRD)

To expand the formation of a ray according to the ratio of a fixed effect, each point must have a fixed concentration fraction or mixing ratio of the components. Thus, appropriate dilutions are used to create a ray for the different effects of the mixture so as to obtain the mixture system toxicity change law.

(2) Equivalent effect concentration ratio (EECR)

The equal effect fixed concentration ratio method is used to examine one special mixture ray among a large number of mixture rays in a system [31]. Based on a mixture with the same effect concentration for each component in the mixture, a series of effect concentration ratios is created, which is then used to obtain different concentration gradients by FRRD expansion. For example, EE10 represents a series where the 96 h-LC_10_ of mixture A is added to the 96 h-LC_10_ of mixture B and expanded.

(3) Direct equipartition ray (EquRay)

**Figure 1:**
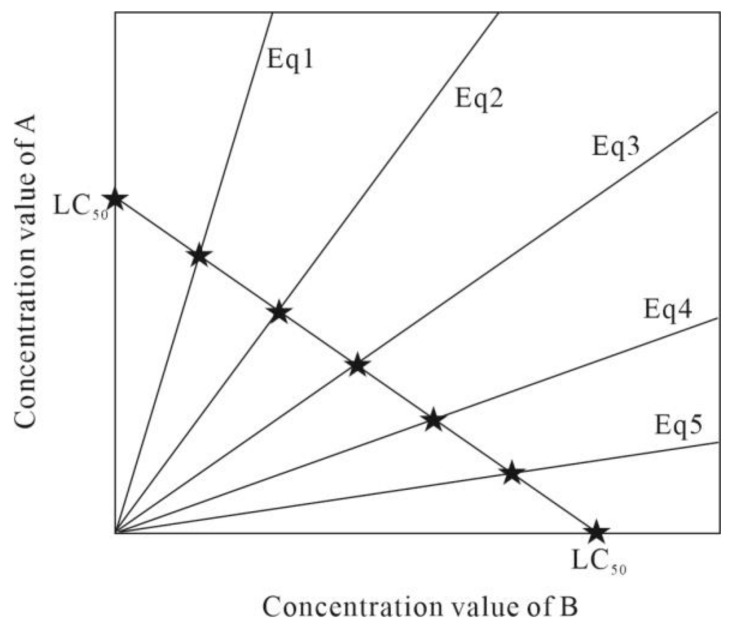
Principle diagram of the direct equipartition ray method.

Combined toxicity depends not only on the concentration levels in the mixture, but also on the mixing ratio, which can affect the toxicity magnitude. The direct equipartition ray method is a way to select some representative mixture concentration points for a binary mixture system reasonably effectively [32], and then expand it by FRRD. This method can systematically design several mixture points to comprehensively characterize the concentration distribution of the binary mixture system and examine the toxicity change pattern of the binary mixture. For example, the schematic diagram shown in Figure 1 details the design of five equalization points, where Eq1 represents the combination of 5/6 of the 96 h-LC_50_ value for mixture A with 1/6 of the 96 h-LC_50_ value for mixture B and expanded. 

#### 2.2.3. Combined Acute Toxicity Prediction

Combined toxicity was predicted based on the concentration addition (CA) and independent action (IA) models [33], and LC_50_ values obtained from the mixture experiments were compared with the predicted LC_50_ values from on each model. If the result was greater than 1, the combined toxicity was antagonistic (the toxic effect of the combined pollutants is less than the sum of the toxic effects of each pollutant alone); if the result is less than 1, the combined toxicity is synergistic (the toxic effect of the combined pollutants is greater than the sum of the toxic effects of each pollutant alone); if the result is equal to 1, the combined toxicity is additive (the toxic effect of the combined pollutants is equal to the sum of the toxic effects of each pollutant alone).

### 2.3. Chemical Analysis

Before and after the experiment, a 4.5 mL sample of each group of solutions was placed into a 5mL centrifuge tube, digested with 0.5 mL HNO_3_ (superior purity), and stored after processing. The samples were analysed for elemental content three times using inductively coupled plasma-mass spectrometry (ICP-MS, X-series 11), which was completed in the National Defense Key Discipline Laboratory of Biotechnology in Uranium Mining and Metallurgy, University of South China.

### 2.4. Data Processing

Origin 2021 (OriginLab, Northampton, UK) and CorelDraw 2021 (Corel, Ottawa, ON, Canada) software was used to create experimental plots. Experimental data were processed and analysed using the MIXTOX toolkit [34] based on the R v3.4.2 (Ross Ihaka and Robert Gentleman, Auckland, New Zealand) software platform. This MIXTOX toolkit integrated concentration–response curve (CRC) fitting, experimental design, and mixed toxicity prediction. Data processing included model fitting for 24, 48, 72 and 96 h CRC for single toxicity experiments with U, Cd and Cu to obtain LC_x_ for each lethal concentration. These models were fitted to the 96 h CRC for the combined U, Cd and Cu toxicity experiments, and the toxic effects were predicted and analysed.

The MIXTOX toolkit provides a total of 13 corresponding functions, and 6 S-type nonlinear functions (Table 1) were selected for fitting according to the response range (0–1) of this experiment, and the fitted optimal term was selected. The criteria for selecting the optimal term for the function fit were the corrected coefficient of determination ($R_{adj}^{2}$, Equation (1)) and the mean absolute error (MAE, Equation (2)). $R_{adj}^{2}\;$was used to correct for *R*^2^ degrees of freedom for the coefficient of determination, and MAE was used to measure the prediction or the amount of closeness of the prediction to the final result: the higher the value for $R_{adj}^{2}$ and the lower the value for MAE, the better the fitted function. The corresponding residual distribution of a single fit CRC is shown in a normal distribution quantile-quantile plot (Q-Q plot). An observation confidence interval (OCI, Equation (3)) is additionally added to the CRC fitting process, which is constructed by adding the uncertainty of the observed data to the uncertainty of the nonlinear function fit describing the experimental data [35,36].

(1) $$R_{adj}^{2}=1-\frac{(n-1)\sum {\left(y_{i}-{\hat{y\;}}_{i}\right)}^{2}}{(n-m)\sum {{(y}_{i}-\;{\bar{y}}_{i})}^{2}}$$

where, n is the number of observations; m is the number of parameters for the fitted model; n−m is the degrees of freedom for the independent variables for calculating the total sum of squares; $y_{i}$ is the response value; ${\hat{y}}_{i}$ is the fitted value; and ${\bar{y}}_{i}$ is the response mean.

(2) $$\mathrm{MAE}=\frac{\sum \left|y-\hat{y\;}\right|}{n}$$

(3) $$\mathrm{OCI}=\hat{y\;}{\;\pm \;t}_{(n-m,\;\frac{\alpha }{2})}\cdot \sqrt{s^{2}{+vCv}^{T}}$$

where, α is the significance level; t is the critical value at degrees of freedom and α; C is the covariance matrix of the parameter estimates obtained from the nonlinear fit; v is the row vector; and $v^{T}$ is the column vector.

The combined toxicity prediction uses the concentration additive model CA with the independent action model IA. The CA model assumes that chemicals have the same mode of action. The model is concentration-based, sums the toxicity of chemicals with similar mode of action, and reflects their relative toxicity in proportion to each other. The commonly used equation (Equation (4)) is as follows:

(4) $${EC}_{x,mix}=(\overset{n}{\underset{i=1}{\sum }}\frac{p_{i}}{{EC}_{x,i}}{)}^{-1}$$

where, $p_{i}$ is the ratio of the concentration of component i to the concentration of the mixture in the compound; ${EC}_{x,i}$ is the concentration of component i when it reaches the x effect; and ${EC}_{x,mix}$ is the concentration of multiple components when they reach the x effect.

In addition, the IA model assumes that chemicals affect the organism through different modes of action, so that their effects are statistically independent of each other. The commonly used equation (Equation (5)) is as follows:

(5) $${E(c}_{mix}{)=E(c}_{1}{+\ldots +c}_{n})=1-\overset{n}{\underset{i=1}{\prod }}[1-{E(c}_{i})]$$

where, $c_{mix}$ denotes the sum of the concentrations of each component in the mixture; $c_{i}$ denotes the concentration of component i when the mixture reaches the x% effect; and ${E(c}_{i})$ denotes the concentration of component i when it acts alone to reach the x% effect.

## 3. Results of Single Acute Toxicity Test

The solution was kept constant during the test to avoid errors caused by the introduction of other media into the experiment. A single toxicity experiment concentration gradient was designed based on the results for U, Cd and Cu in pilot experiments. Table 2 shows the specific values for the single acute toxicity experiment concentration design.

**Figure 2:**
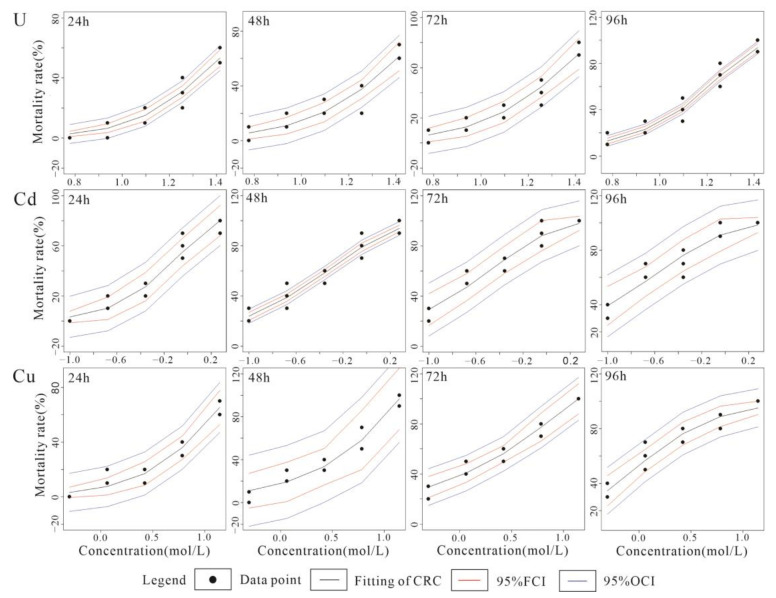
CRC fit for U, Cd and Cu in *C. vidua* single acute toxicity experiments.

**Figure 3:**
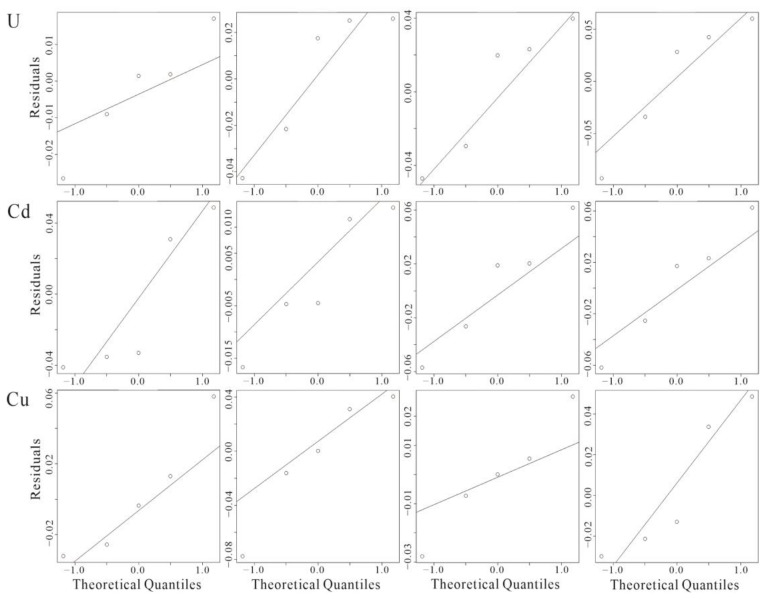
Q-Q diagrams of the fitted normal distributions for U, Cd and Cu in single acute toxicity experiments with *C*. *vidua*.

The results of the hydration parameters and CRC fit data for the single acute toxicity experiments for U, Cd and Cu with *C. vidua* are listed in Table 3 and Table 4, respectively, and the CRC fit plots and normal distribution Q-Q plots are shown in Figure 2 and Figure 3, respectively.

Table 3 shows that the hydration parameters before and after the experiment were stable and within the appropriate range for the survival of the ostracod species. The survival rate of the ostracods in the blank control group was higher than 90%, which was in accordance with the stability requirements of toxicological biomaterials. The results of the single CRC fits for U, Cd and Cu and *C. vidua* for each time period are presented in Table 4. The best-fit model was selected based on $R_{adj}^{2}$ and MAE, and the three effect concentration values of LC_10_, LC_30_ and LC_50_ with 95% confidence intervals are presented. As seen from Table 4, the minimum value for the correction coefficient of determination $R_{adj}^{2}$ was 0.952, and the maximum value for the mean absolute error MAE was 0.037, indicating a high quality CRC fit. The LC_50_ values for U at 24, 48, 72, and 96 h were 24.723 mg·L^−1^, 21.998 mg·L^−1^, 19.481 mg·L^−1^, and 14.244 mg·L^−1^, respectively, and decreased with increasing exposure time, and the maximum and minimum difference was approximately 1.7-fold. The LC_50_ values for Cd 24, 48, 72, and 96 h were 0.801 mg·L^−1^, 0.327 mg·L^−1^, 0.232 mg·L^−1^ and 0.158 mg·L^−1^, respectively. The LC_50_ values for Cu at 24, 48, 72 and 96 h were 9.313 mg·L^−1^, 4.830 mg·L^−1^, 1.964 mg·L^−1^ and 0.898 mg·L^−1^, respectively. According to the Global Harmonized System of Classification and Labelling of Chemicals (GHS) [37], the acute aquatic environmental toxicity of U, Cd and Cu are graded according to the following scale: very high toxicity, 96 h-LC_50_ (mg·L^−1^) ≤ 1; high toxicity, 1 < 96 h-LC_50_ (mg·L^−1^) ≤ 10; moderate toxicity, 10 < 96 h-LC_50_ (mg·L^−1^) ≤ 100; and low toxicity, 96 h-LC_50_ > 100. Both Cd and Cu had very high toxicity, whereas U had moderate toxicity.

Figure 2 shows the CRC fit for U, Cd and Cu in *C. vidua* single acute toxicity experiments. As the exposure time increased, the Cd and Cu fitting curves gradually changed from a threshold type to asymptotic lines, whereas the U fitting curves were predominantly the threshold type for all time periods. Throughout the experiment, a strong dose-effect relationship between biological mortality and solution concentration was characterized in each group, mainly showing a significant increase in biological mortality with increasing solution concentrations. During the early stages, Cd and Cu could cause high mortality at high concentration levels and U exhibited weaker toxicity, but U toxicity increased significantly in the late stage of the experiment and mortality increased rapidly. Figure 3 shows Q-Q plots of the fitted normal distribution for U, Cd and Cu, which basically conformed to a normal distribution except for U at 24 h and Cd at 72 h. Large deviations at low concentrations were observed for U at 96 h and Cu at 48 h, and Cu at 24 h showed large deviations at high concentrations.

## 4. Results and Data Simulation of Combined Acute Toxicity Experiments

The specific concentration design ratios for combined acute toxicity experiments with *C. vidua* are shown in Table 5. The results of the combined toxicity experiments were fitted by CRC using the six models shown in Table 1. Toxicity prediction was carried out using the CA and IA models, and the data from both were compared to determine the toxicity effects in each group of experiments.

### 4.1. U-Cd Combined Acute Toxicity Test

**Figure 4:**
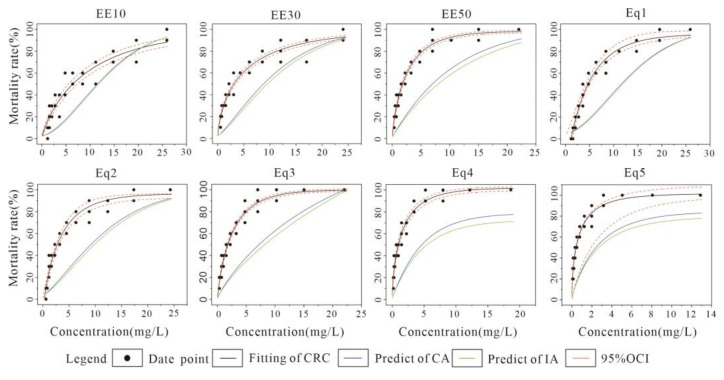
Results from *C*. *vidua* U-Cd combined acute toxicity experiments with fitted CRC and CA and IA model predictions.

The results of CRC fitting and the fitting plots for the combined U-Cd acute toxicity assay are listed in Table 6 and Figure 4, respectively. The minimum value of $R_{adj}^{2}$ for each combination in Table 6 was 0.967, and the maximum value of MAE was 0.034, indicating a good quality CRC fit. Compared to the commonly used two-parameter model, the three-parameter model (GL and BCW) performed better in the fitting effect. The EECR combination showed a decrease in LC_50_ values as the concentration level increased (EE10–EE50), with a minimum LC_50_ value of 1.791 mg·L^−1^ for the EE50 group. An overall increase in the mortality of *C. vidua* was observed with the high toxic effect mixture, causing some mortality even at the lowest concentration levels in the FRRD extension. The EquRay combination showed a decrease in LC_50_ values and an increase in toxicity as the percentage of Cd increased (Eq1–Eq5). As the most toxic substance, Cd also caused more toxic effects on *C. vidua* in the higher percentage combinations. Both CA and IA models showed that the predicted LC_50_ values were higher than the measured values, and the ratio of the measured LC_50_ values divided by the model predicted LC_50_ values was less than 1. It was found that the toxic effects were synergistic, and the predictions of the CA model were closer to the measured values.

Figure 4 shows the fitted CRC, and CA and IA model predictions for the combined acute toxicity experimental results for U-Cd. Except for EE10, all combinations showed a higher experimental fitted CRC than the model predicted CRC, and the toxic effects showed a synergistic effect. The synergistic effect of EE30, EE50, Eq1, Eq2 and Eq3 gradually increased first and then decreased as concentrations increased, then approached experimental toxicity at high concentration levels. As the concentration of Eq4 and Eq5 gradually increased, the degree of synergy continued to increase and no longer decreased. The toxic effects of EE10 appeared to be synergistic as the concentration gradually increased, but after reaching a certain concentration, the toxic effects changed to additive and eventually showed a slight antagonistic effect. The CA model predictions were more consistent with the experimental situation than the IA model, but there were some differences.

### 4.2. U-Cu Combined Acute Toxicity Test

**Figure 5:**
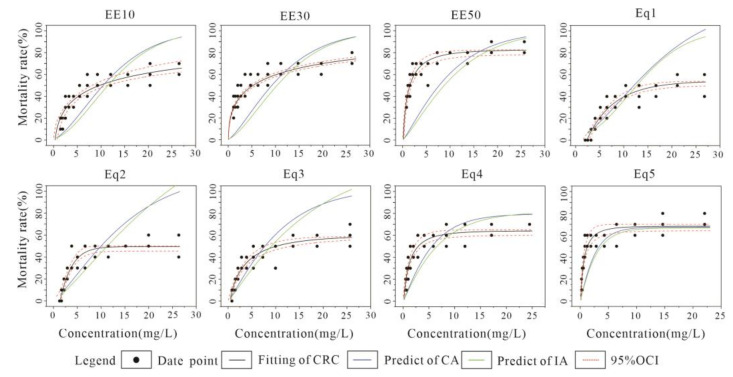
Results from *C. vidua* U-Cu combined acute toxicity experiments with fitted CRC and CA and IA model predictions.

The results of CRC fitting and the fitting plots for the combined U-Cu acute toxicity assay are listed in Table 7 and Figure 5, respectively. For each combination in Table 7, the minimum value of $R_{adj}^{2}$ was 0.922 and the maximum value of MAE was 0.034. Although the quality of CRC fit was negative for the combinations of EE50 and Eq5, it was a good for the rest of the combinations. A three-parameter model was mainly used for CRC fitting, and only EE30 required a two-parameter model. The pattern for the EECR combination was consistent for U-Cd: as the concentration levels increased, the LC_50_ value decreased and toxicity increased, where the minimum value for LC_50_ was 1.422 mg·L^−1^. With an increase in Cu percentage in the EquRay combination, the LC_50_ value decreased and changed more obviously, and the maximum and minimum values differed approximately 18-fold. The CA and IA models both predicted LC_50_ values that were higher than the measured values when the mixing mode was dominated by EECR, and the toxicity effects were synergistic. Among them, the difference between the predicted and measured values for the EE10 combination was smaller and within the confidence interval of the OCI, and the prediction results were better. When the mixture was dominated by EquRay, the measured LC_50_ values for Eq1, Eq2 and Eq3 were higher than the predicted values, which showed antagonistic effects, and the measured LC_50_ values for Eq4 and Eq5 were lower than the predicted values, which showed synergistic effects. Thus, the toxic effects changed from antagonistic to synergistic as the proportion of Cu in the mixture gradually increased.

Figure 5 shows the results of the combined U-Cu acute toxicity experiments with fitted CRC and model predictions. The patterns for the remaining seven combinations, except Eq5, showed that the toxic effects were synergistic at low concentrations, and the toxic effects shifted from synergistic to additive and then antagonistic as the concentration level gradually increased. Such a pattern was similar to the EE10 combination for U-Cd, but the change occurred at low and medium concentration levels. The prediction of the CA model was better at low concentration levels and the IA model was better at high concentration levels. The prediction of both models for Eq5 was good overall, with a weak synergistic effect at low concentrations, and the predicted values were almost the same as the measured values at high concentration levels, showing an additive effect, and the prediction of the CA model was better.

### 4.3. Cd-Cu Combined Acute Toxicity Test

The minimum value for $R_{adj}^{2}$ for each combination in Table 8 was 0.955 and the maximum value of MAE was 0.054. The patterns for the EECR combinations were consistent with the results for the above two experimental groups, so as the concentration level gradually increased, there was a decrease in LC_50_ value and an increase in toxicity. The minimum value for LC_50_ in the EE50 group was 0.437 mg·L^−1^ for the three combined experiments. For the EquRay combinations, the LC_50_ values increased as the proportion of Cu fraction gradually increased, but the increase was smaller and slower, and the LC_50_ in the Eq5 group reached a maximum value of 0.135 mg·L^−1^. The IA model predicted values closer to the measured values and had better effects.

**Figure 6:**
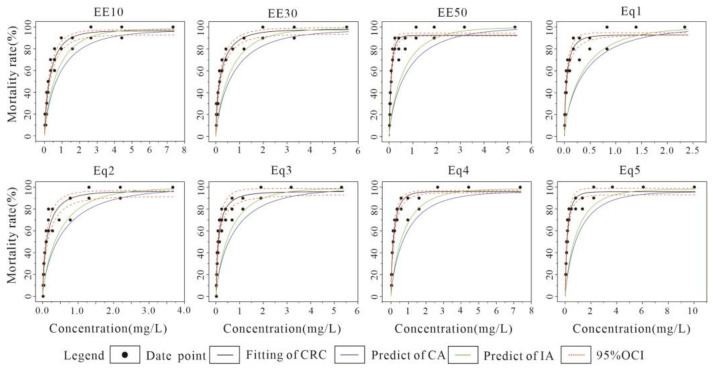
Results for *C*. *vidua* Cd-Cu combined acute toxicity experiments with fitted CRC and CA and IA model predictions.

Figure 6 shows the fitted CRC and the predictions of the CA and IA models compared to the measured values from the combined acute toxicity experiments with Cd-Cu. The overall model predictions were good, and the toxicity effects for the different combinations were divided into three patterns: the first toxicity effect varied from synergistic to additive (EE30, Eq3, Eq5); the second toxicity effect varied from synergistic to additive and then antagonistic (EE50, Eq1); the third effect predicted by the CA model varied from synergistic to additive and then antagonistic (EE50, Eq1). However, the IA model predicted that only additive effects occurred at high concentration levels and did not change to antagonistic effects (EE10, Eq2, Eq4). The prediction of the CA model was better at low concentration levels, the IA model was better at high concentration levels.

## 5. Discussion

### 5.1. Discussion of Single Acute Toxicity Experiments

The results of single acute toxicity tests have showed that Cd and Cu are toxic within a short period of time with a maximum lethal concentration rated as highly biohazardous, whereas U, as a radionuclide and a heavy metal, was both radiologically and chemically toxic [38]. In acute toxicity experiments, U is almost exclusively chemically toxic and hardly shows radiotoxicity in the form of uranyl ions that irreversibly bind to sulfhydryl groups of protein amino acid residues (antioxidant enzymes, DNA repair enzymes and membrane channel proteins) causing their inactivation, and thus, oxidative stress [39,40,41]. The chemical toxicity of U mainly occurs after 48 h, and the maximum lethal dose is reached at 96 h. This is significantly different from Cd and Cu, two heavy metals with obvious chemical toxicity (showing toxicity in a short time), but biological toxicity is not obvious for the first 48 h. Both Cd and Cu are highly toxic and can have serious biological effects at low concentrations, inducing oxidative stress, apoptosis and immunotoxicity, where the mechanism of toxicity is related to the MAPK (mitogen-activated protein kinase) signalling pathway [42,43,44].

Table 9 summarises the toxicity of U, Cd and Cu for various organisms after a single exposure. During the present experiment, the 96 h LC_50_ values for Cd, Cu and U against *C. vidua* were 0.158 mg·L^−1^, 0.898 mg·L^−1^ and 14.244 mg·L^−1^, respectively, and according to the calculated toxicity factor (ratio of 96 h-LC_50_ values) [45], Cd was 5.7 and 90 times more toxic than Cu and U, respectively. This result is similar to Charles et al. [46], which determined Cu was approximately 13 times more toxic than U for duckweed *Lemna aequinoctialis*, and Cu was about 14 times more toxic than U for zebrafish *Brachydanio rerio* [47,48]. The toxicity of U, Cd and Cu for *C. vidua* when acting alone can be ranked from largest to smallest: Cd > Cu > U.

### 5.2. Discussion of Combined Acute Toxicity Experiments

#### 5.2.1. Toxicity Evaluation of Combined Effects

The LC_50_ values and synergistic ratios for binary mixtures of U, Cd and Cu for *C. vidua* are shown in Table 10.

Toxicity evaluation models use synergistic ratios (SRs) [45] to measure the combined effect toxicity. The results for SR values are derived from the following equation (Equation (6)):

SR = S 96 h-LC_50_/C 96 h-LC_50_ (6)

where, S 96 h-LC_50_ denotes the values for single heavy metals; C 96 h-LC_50_ denotes the values for combined heavy metals.

SR = 1 describes an additive effect; SR < 1 describes an antagonistic effect; SR > 1 describes a synergistic effect.

In the combined toxicity experiments with *C. vidua*, it was observed that the mixed solutions of U-Cd in any ratio were more toxic than U on its own. The SR values for U were all larger than 1, showing a synergistic effect. On the other hand, the SR values for Cd in mixed solutions in any ratio was smaller than Cd on its own, and the SR values for Cd were all smaller than 1, showing an antagonistic effect. One of the main bioavailable forms of U is the uranyl cation UO_2_^2+^ [55]. Competition between UO_2_^2+^ and Cd^2+^ for one transporter depends on their concentration, affinity for the transporter and internalization kinetics, and differences in the concentration levels of U and Cd can lead to a reduction in Cd competition [56]. In this study, the concentration of U was at least 18 times higher than Cd, with an average design concentration of approximately 190 times, and this high concentration difference led to a reduction in the toxicity produced by Cd in the presence of U. Therefore, the toxicity of U-Cd falls between the toxicity of U and Cd.

When U-Cu was configured in a mixed solution according to the ratio of Eq1 or Eq2, the SR value for U was smaller than 1, which showed antagonism, and the rest showed synergism, whereas the SR value for Cu in this mixture configured in any ratio was smaller than 1, which showed antagonism. The antagonism between the two metals may be due to the fact that the metals in solution have a similar binding affinity for the cell surface, so one metal reduces the available binding sites for the other metal [57]. Since the cell surface binding affinity of U is slightly higher than that of Cu, its preferential binding to the cell surface leads to fewer effective binding sites for Cu, thus reducing its toxicity. Charles et al. [46] found in an ecotoxicological study of U and Cu with *Lemna aequinoctialis* that the mixture of U and Cu inhibited the of growth rate was reduced and the combined effect of the two showed an antagonistic effect. This result is similar to that for Eq1 and Eq2 in the U-Cu combination experiment.

Cd-Cu presented a synergistic effect on *C. vidua* except when the SR value for Cd was smaller than 1 for the EE10 solution, which showed an antagonistic effect. The SR value for Cu in solution configured with any ratio was always higher than 1, showing a synergistic effect. The dominant process for combined Cd-Cu toxicity is competition for metal-to-metal binding biotic ligands (BLs). As long as Cu is maintained at sublethal concentrations, mortality due to Cd decreases because the less toxic Cu prevents the more toxic Cd from binding to BL. However, when Cu concentrations reach a single LC_50_, at which point Cu also begins to produce lethal toxicity, mortality begins to increase and the combined effect manifests itself as a synergistic effect [43,58,59].

#### 5.2.2. Toxicity Effect Change Pattern

In the combined toxicity experiments, the level of concentration directly affected the magnitude of toxicity, and in the same way, any change in the mixing ratio could also lead to changes in toxicity. When metals are mixed to produce toxicity, the ratio of individual metal concentrations can change the toxicity of the composite as a whole. For example, in this experiment the toxicity ratio for U-Cd increased from 5:1 to 4:2 for Eq1 and Eq2 and produced a 1.7-fold increase in toxicity. A comparison of the same toxicity ratios (Eq1 and Eq2) revealed a 1.1-fold increase in toxicity for U-Cu and a 2.7-fold decrease for Cd-Cu. This toxicity pattern correlated with the single toxicity of each element, so when the proportion of a metal with relatively greater toxicity was increased, its compound toxicity also increased, and vice versa.

#### 5.2.3. Model Prediction of Combined Toxicity

The MIXTOX modelling package based on the R language is a stepwise statistical procedure that evaluates whether data obtained from combined toxicity experiments conform to concentration additive (CA) or independent action (IA) reference models, and the results can exhibit synergistic, antagonistic, or compound interactions [60,61]. Varano et al. [62] found that the CA model showed significant antagonistic effects of fluoxetine and propranolol for *Daphnia magna* in acute combined toxicity experiments. Gong et al. [63] studied the toxicity of single and combined mixtures of Y, La and Ce on wheat (*Triticum aestivum*) using both the CA and IA reference models to perform mixed toxicity analysis, and satisfactory model predictions were obtained.

Both the CA and IA models in this study showed that when U and Cd were used in a mixture, the whole predicted CRC was mainly synergistic, where the degree of synergy was obvious for the Eq4 and Eq5 combinations, and only weak antagonism for the high concentration gradient of the EE10 combination. The CRC predicted by the CA model was closer to the measured CRC, with U operating with the same mechanism of action as Cd. When U and Cu were used in the mixture, the CRC was predicted to change at different gradients, often showing synergistic effects at low concentration gradients that transformed into antagonistic effects at high concentration gradients. At this point, the CA and IA models also transformed, where the combined mode of action for the low concentration gradient was CA and the combined mode of action for the high concentration gradient was IA. This result is different from a simple synergistic/antagonistic effect, where there is a dose level-dependent deviation (DL) because the two reference models deviate differently at low versus high dose levels [64]. This conclusion is similar to that of Margerit et al. [56], whose study on the combined toxicity of U and Cd for the nematode Caenorhabditis elegans found that low dose levels were synergistic and high dose levels were antagonistic. When Cd was mixed with Cu, the predicted results were overall similar to those for the U-Cd mixture, namely, synergistic effects and better IA model predictions, with both metals producing toxicity in a mutually independent form. Therefore, the model predictions demonstrated that the effects of U-Cd on ostracods occurred in a concentration-additive manner; the mode of action for U-Cu transformed between low and medium concentrations and high concentrations; and the toxicity of Cd-Cu mainly affected the ostracods with an independent mode of action.

## 6. Conclusions

There has been increasingly widespread concern about radioactive and heavy metal contamination from uranium mine mining. In this study, we explored the ecotoxic effects of three common U, Cd and Cu elements of uranium mine contamination on the ostracod *C. vidua*. The main conclusions are as follows:

(1) The 96-h LC_50_ values for the radionuclide U and the heavy metals Cd and Cu for *C. vidua* were 14.244 mg·L^−1^, 0.158 mg·L^−1^ and 0.898 mg·L^−1^, respectively. On the basis of the 96-h LC_50_ (the smaller the value, the greater the toxicity), the toxicity of the three contaminating elements for *C. vidua* was Cd > Cu > U. At the maximum lethal concentration, the toxicity of Cd and Cu could be detected in a short period of time. In contrast, U, as a radionuclide, was weakly chemically toxic and its toxicity was not detectable in a short period of time, but it still caused maximum mortality in *C. vidua* later in the experiment.

(2) The combined toxicity experiments investigated the compound effects of U, Cd and Cu in binary mixtures, and the compound toxicity change pattern was analysed by replacing the proportion of one part of the binary mixture. U-Cd and U-Cu combination experiments showed compound toxicity for a single weakly toxic component and a highly toxic component. Different combinations of toxicity patterns showed uniform characteristics, such that when the proportion of a metal with relatively higher toxicity was increased, the compound toxicity also increased, and vice versa.

(3) When the experimentally fitted CRC results were compared with the predicted results from the combined toxicity model, it was shown that the combined toxicity effect of U-Cd was mainly synergistic that could be described by the concentration addition model; the combined toxicity effect of Cd-Cu was also mainly synergistic, but the independent action model was more applicable; and the combined toxicity effect of U-Cu changed at different concentration levels, with a synergistic effect at low and medium concentration levels, and an antagonistic effect was present at high concentration levels, so the best model changed from CA to IA.

## Figures and Tables

**Table 1:** Six S-type nonlinear functions and their corresponding inverse functions.

Function Name	f(x)	${x=f}^{-1}\left(Y\right)$
Hill	f(x)=αx/(β+x)	x=βY/(α−Y)
Logit	$f(x)=(1+\exp(-\alpha -{\beta \mathrm{lg}(x)))}^{-1}$	${x=10}^{((\mathrm{lg}(Y/(1-Y))/\mathrm{lg}e)-\alpha )/\beta }$
Weibull	f(x)=1−exp(−exp(α+βlg(x)))	${x=10}^{(\mathrm{lg}(-\mathrm{lg}(1-{Y)/\mathrm{lg}e)/\mathrm{lg}}_{e-\alpha })/\beta }$
Box–Cox–Weibull (BCW)	$f(x)=1-\exp(-{\exp(\alpha +\beta ((x}^{\gamma }-1)/\gamma )))$	$x=((\gamma /\beta )(\mathrm{lg}(-\mathrm{lg}(1-{Y)/\mathrm{lg}e)/\mathrm{lg}}_{e-\alpha }{)+1)}^{1/\gamma }$
Box–Cox–Logit (BCL)	$f(x)=(1+\exp(-\alpha -{\beta ((x}^{\gamma }-{1)/\gamma )))}^{-1}$	$x=((\gamma /\beta )(-\mathrm{lg}(\frac{(1-Y)}{Y}{)/\mathrm{lg}}_{e-\alpha }{+1))}^{1/\gamma }$
Generalised Logit (GL)	$f(x)=1/(1+\exp(-\alpha -{\beta \mathrm{lg}(x)))}^{\gamma }$	${x=10}^{((-\mathrm{lg}((\frac{1}{Y}{)}^{\frac{1}{\gamma }}-{1)/\mathrm{lg}}_{e-\alpha })/\beta )}$

**Table 2:** *C. vidua* single acute toxicity experimental concentration design values.

Concentration Group	U/mg·L^−1^	Cd/mg·L^−1^	Cu/mg·L^−1^
1	6	0.1	0.5
2	8.66	0.21	1.15
3	12.49	0.44	2.65
4	18.02	0.91	6.09
5	26	1.9	14

**Table 3:** Solution hydration parameters before and after *C. vidua* experiments.

Element	Time	pH Value	Temperature/ °C	Dissolved Oxygen/ mg·L^−1^	Hardness/ mg·L^−1^
U	Before	7.5 ± 0.1	25 ± 0.1	6.5 ± 0.2	155 ± 1
	After	7.5 ± 0.1	25 ± 0.1	5.4 ± 0.2	155 ± 1
Cd	Before	7.6 ± 0.1	25 ± 0.1	5.9 ± 0.2	147 ± 1
	After	7.5 ± 0.1	25 ± 0.1	5.5 ± 0.2	147 ± 1
Cu	Before	7.4 ± 0.1	25 ± 0.1	6.0 ± 0.2	154 ± 1
	After	7.4 ± 0.1	25 ± 0.1	5.5 ± 0.2	154 ± 1

**Table 4:** CRC fit data for *C. vidua* single acute toxicity assays.

Element	Time/h	Function	α	β	γ	$R_{adj}^{2}$	MAE	Concentration/mg·L^−1^		
								LC_10_	LC_30_	LC_50_
U	24	Logit	−8.161	5.858	/	0.991	0.011	10.424 (8.901–11.745)	17.721 (16.649–18.791)	24.723 (23.245–26.294)
	48	Weibull	−6.331	4.443	/	0.976	0.026	8.286 (5.539–10.479)	15.590 (13.651–17.471)	21.998 (19.874–24.211)
	72	Weibull	−6.339	4.631	/	0.974	0.031	7.636 (4.698–9.846)	14.001 (11.938–15.975)	19.481 (17.454–21.591)
	96	GL	−20.611	15.376	0.236	0.999	0.005	5.105 (4.429–5.731)	10.236 (9.789–10.673)	14.244 (13.794–14.691)
Cd	24	Logit	0.358	3.732	/	0.976	0.037	0.206 (0.078–0.311)	0.475 (0.352–0.610)	0.801 (0.642–1.000)
	48	Weibull	0.515	1.817	/	0.997	0.010	0.030 (0.021–0.038)	0.141 (0.123–0.160)	0.327 (0.300–0.355)
	72	Weibull	0.833	1.892	/	0.967	0.036	0.023 (0.002–0.052)	0.103 (0.057–0.161)	0.232 (0.167–0.314)
	96	Weibull	0.947	1.644	/	0.952	0.037	0.011 (0.001–0.031)	0.062 (0.028–0.111)	0.158 (0.104–0.234)
Cu	24	Weibull	−2.694	2.402	/	0.973	0.026	1.530 (0.569–2.531)	4.926 (3.604–6.376)	9.313 (7.462–11.454)
	48	GL	−30.261	26.592	0.057	0.964	0.033	0.426 (0.001–1.569)	2.236 (0.795–4.104)	4.830 (2.601–7.482)
	72	GL	−87.563	81.627	0.011	0.990	0.013	0.030 (0.003–0.100)	0.523 (0.266–0.894)	1.964 (1.429–2.608)
	96	Logit	0.115	2.493	/	0.975	0.029	0.118 (0.030–0.214)	0.411 (0.269–0.586)	0.898 (0.685–1.177)

**Table 5:** Concentration ratio design for *C*. *vidua* combined acute toxicity assay.

Concentration Design	Concentration Ratio					
	U	Cd	U	Cu	Cd	Cu
EE10	0.9978	0.0022	0.9774	0.0226	0.0853	0.9147
EE30	0.9939	0.0061	0.9653	0.0347	0.1462	0.8538
EE50	0.9890	0.0110	0.9434	0.0566	0.1560	0.8440
Eq1	0.9978	0.0022	0.9881	0.0119	0.4802	0.5198
Eq2	0.9945	0.0055	0.9709	0.0291	0.2699	0.7301
Eq3	0.9890	0.0110	0.9434	0.0566	0.1560	0.8440
Eq4	0.9783	0.0217	0.8928	0.1072	0.0846	0.9154
Eq5	0.9475	0.0525	0.7692	0.2308	0.0356	0.9644

**Table 6:** Fitting data and model predictions for the combined U-Cd acute toxicity experiments with *C*. *vidua*.

Combination	Function	α	β	γ	$R_{adj}^{2}$	MAE	LC_30_ (mg·L^−1^)			LC_50_ (mg·L^−1^)		
							Actual Measured Value (Confidence Interval)	CA	IA	Actual Measured Value (Confidence Interval)	CA	IA
EE10	Weibull	−1.872	1.861	/	0.967	0.034	2.831 (2.324–3.384)	7.523	7.762	6.439 (5.693–7.257)	11.9	12.1712
EE30	Weibull	−1.118	1.510	/	0.989	0.02	1.142 (0.993–1.303)	5.149	5.661	3.147 (2.887–3.425)	9.252	0.073
EE50	GL	−2.755	4.097	0.368	0.994	0.016	0.764 (0.681–0.852)	3.668	4.127	1.791 (1.645–1.945)	7.201	8.163
Eq1	GL	0.680	3.080	10.667	0.992	0.019	2.942 (2.732–3.163)	8.303	8.39	4.528 (4.217–4.870)	12.637	12.727
Eq2	BCW	−1.523	1.394	−0.359	0.984	0.028	1.458 (1.282–1.648)	7.17	7.472	2.677 (2.375–3.021)	11.547	11.907
Eq3	Weibull	−0.847	1.900	/	0.995	0.016	0.800 (0.725–0.877)	3.448	3.884	1.789 (1.679–1.905)	6.863	7.818
Eq4	GL	−5.722	8.191	0.123	0.989	0.021	0.315 (0.246–0.394)	3.896	4.376	1.020 (0.898–1.150)	7.542	8.502
Eq5	BCW	0.141	0.636	0.130	0.996	0.01	0.121 (0.103–0.141)	3.668	4.127	0.430 (0.393–0.469)	7.201	8.163

Note: the 95% observation confidence interval is 95% OCI.

**Table 7:** Fitting data and model predictions for the combined U-Cu acute toxicity experiments with *C*. *vidua*.

Combination	Function	α	β	γ	$R_{adj}^{2}$	MAE	LC_30_ (mg·L^−1^)			LC_50_ (mg·L^−1^)		
							Actual Measured Value (Confidence Interval)	CA	IA	Actual Measured Value (Confidence Interval)	CA	IA
EE10	GL	5.711	1.136	618.052	0.948	0.029	2.923 (2.427–3.508)	6.643	7.643	8.958 (7.482–10.779)	10.662	11.868
EE30	Logit	−0.993	1.431	/	0.983	0.013	1.265 (1.086–1.465)	5.323	6.284	4.944 (4.563–5.357)	9.054	10.43
EE50	BCW	−0.607	0.752	−0.544	0.922	0.033	0.611 (0.451–0.822)	4.231	5.017	1.422 (1.184–1.729)	7.571	8.903
Eq1	BCW	−12.943	18.087	−1.417	0.982	0.019	6.771 (6.196–7.440)	7.573	8.473	19.939 (15.491–29.279)	11.69	12.671
Eq2	BCW	−6.677	10.999	−1.732	0.975	0.020	3.556 (3.211–3.980)	6.936	7.916	18.309 (9.540–25.328)	10.995	12.137
Eq3	BCW	−2.174	1.510	−0.660	0.972	0.021	2.856 (2.472–3.311)	3.68	4.341	10.646 (8.632–13.504)	6.762	7.993
Eq4	BCW	−0.976	0.807	−0.709	0.958	0.023	0.936 (0.800–1.099)	4.335	5.142	2.951 (2.335–3.875)	7.718	9.063
Eq5	BCW	−0.472	0.522	−0.749	0.938	0.034	0.455 (0.371–0.561)	4.231	5.017	1.244 (0.940–1.748)	7.571	8.903

Note: the 95% observation confidence interval is 95% OCI.

**Table 8:** Fitting data and model predictions for combined Cd-Cu acute toxicity experiments with *C*. *vidua*.

Combination	Function	α	β	γ	$R_{adj}^{2}$	MAE	LC_30_ (mg·L^−1^)			LC_50_ (mg·L^−1^)		
							Actual Measured Value (Confidence Interval)	CA	IA	Actual Measured Value (Confidence Interval)	CA	IA
EE10	GL	1.195	2.764	0.745	0.985	0.027	0.115 (0.094–0.138)	0.276	0.238	0.258 (0.217–0.307)	0.638	0.523
EE30	GL	1.096	2.902	0.457	0.982	0.026	0.054 (0.042–0.069)	0.237	0.206	0.153 (0.125–0.186)	0.556	0.457
EE50	BCW	1.020	0.263	−0.497	0.984	0.030	0.041 (0.035–0.047)	0.224	0.196	0.075 (0.065–0.086)	0.529	0.437
Eq1	GL	4.065	1.798	3.531	0.978	0.034	0.017 (0.014–0.021)	0.331	0.287	0.038 (0.031–0.047)	0.747	0.625
Eq2	Logit	2.187	2.216	/	0.955	0.054	0.042 (0.029–0.058)	0.322	0.278	0.103 (0.079–0.134)	0.729	0.606
Eq3	GL	9.826	1.732	2195.314	0.979	0.035	0.045 (0.037–0.055)	0.140	0.130	0.095 (0.079–0.116)	0.344	0.303
Eq4	Hill	0.124	1.078	/	0.976	0.034	0.056 (0.044–0.069)	0.194	0.172	0.124 (0.105–0.146)	0.464	0.389
Eq5	GL	3.111	2.234	2.568	0.987	0.023	0.068 (0.059–0.078)	0.224	0.196	0.135 (0.118–0.154)	0.529	0.437

Note: the 95% observation confidence interval is the 95% OCI.

**Table 9:** Acute toxicity data for each single biological exposure to U, Cd and Cu.

Phylum	Species	U	Cd	Cu	Reference
Magnoliophyta	*Lemna aequinoctialis*	3.2 μmol·L^−1^		0.25 μmol·L^−1^	[46]
Vertebrate	*Brachydanio rerio*	3.05 mg·L^−1^	3.8 mg·L^−1^	212 μg·L^−1^	[47,48]
	*Melanotaenia nigrans*	2.16 mg·L^−1^		135 μg·L^−1^	[49]
	*Melanotaenia splendida inornata*	3.94 mg·L^−1^		168 μg·L^−1^	[49]
Mollusca	*Velesunio angasi*	941 μg·L^−1^	184 μg·L^−1^	10.4 μg·L^−1^	[49]
Arthropoda	*Cypridopsis vidua*	14.2 mg·L^−1^	158 μg·L^−1^	898 μg·L^−1^	Present study
	*Chironomus dilutus*	33.5 mg·L^−1^			[50]
	*Hyalella azteca*	8.2 mg·L^−1^	17.5 μg·L^−1^	912 μg·L^−1^	[50,51]
	*Eurytemora affinis* (Males)		127.8 μg·L^−1^	25.0 μg·L^−1^	[52]
	*Eurytemora affinis* (Females)		90.0 μg·L^−1^	38.0 μg·L^−1^	[52]
	*Heterophoxus videns*		1.95 mg·L^−1^	1.30 mg·L^−1^	[53]
	*Stenocypris major*		13.1 μg·L^−1^	25.2 μg·L^−1^	[53]
Echinodermata	*Ophiura flexibilis*		1.95 mg·L^−1^	396 μg·L^−1^	[53]
	*Apostichopus japonicus*		1.57 mg·L^−1^	133 μg·L^−1^	[54]

**Table 10:** Specific LC_50_ values and synergy ratios for binary mixtures of U, Cd and Cu.

Combination	U-Cd			U-Cu			Cd-Cu		
	LC_50_	SR	SR	LC_50_	SR	SR	LC_50_	SR	SR
		U	Cd		U	Cu		Cd	Cu
EE10	6.439	2.212	0.025	8.958	1.590	0.100	0.258	0.612	3.481
EE30	3.147	4.526	0.050	4.944	2.881	0.182	0.153	1.033	5.869
EE50	1.791	7.953	0.088	1.422	10.017	0.632	0.075	2.107	11.973
Eq1	4.528	3.146	0.035	19.939	0.714	0.045	0.038	4.158	23.632
Eq2	2.677	5.321	0.059	18.309	0.778	0.049	0.103	1.534	8.718
Eq3	1.789	7.962	0.088	10.646	1.338	0.084	0.095	1.663	9.453
Eq4	1.02	13.965	0.155	2.951	4.827	0.304	0.124	1.274	7.242
Eq5	0.43	33.126	0.367	1.244	11.450	0.722	0.135	1.170	6.652

## Data Availability

All data are contained in the manuscript.
